# Deciphering the enigma of atypical forms of Alzheimer's disease by employing connectomics in the context of early and late‐phase Amyloid PET and [^18^F]FDG PET Imaging

**DOI:** 10.1002/alz.087349

**Published:** 2025-01-09

**Authors:** Alessio Cirone, Wendy Kreshpa, Sara Garbarino, FEDERICO MASSA, Stefano Raffa, Gianmario Sambuceti, Michele Piana, Andrea Chincarini, Luca Roccatagliata, Antonio Uccelli, Silvia Morbelli, Matteo Pardini

**Affiliations:** ^1^ IRCCS Ospedale Policlinico San Martino, Genova, Liguria Italy; ^2^ Università degli studi di Genova, Genova, Liguria Italy; ^3^ University of Genova, Genova Italy; ^4^ IRCCS Ospedale Policlinico San Martino, Genova Italy; ^5^ IRCCS Ospedale Policlinico San Martino, Genova, Genova Italy; ^6^ Istituto Nazionale di Fisica Nucleare (INFN), Genova, Liguria Italy; ^7^ Università degli studi di Torino, Torino, Piemonte Italy; ^8^ A.O.U. Citta della Salute e della Scienza di Torino, Torino, Piemonte Italy

## Abstract

**Background:**

Alzheimer's disease (AD) is the most common form of dementia, predominantly manifesting as amnestic impairment. However, atypical presentations such as logopenic variant of primary progressive aphasia (lvPPA), posterior cortical atrophy (PCA), corticobasal syndrome (CBS) and frontal AD pose diagnostic challenges. This study presents preliminary data from a retrospective analysis investigating brain functional differences between typical and atypical AD forms. Specifically, a structural connectome, a comprehensive map of anatomical white matter connections in the brain, was employed alongside late‐phase amyloid [^18^F]FBB PET, which resembles focal regional amyloid uptake. Additionally, the research aims to examine whether early‐phase amyloid [^18^F]FBB PET can serve as a surrogate for glucose metabolism throughout various brain regions, similar to [^18^F]FDG PET.

**Method:**

Thirty patients have been recruited, including 13 with atypical AD (median age 70; median MMSE 21) and 17 with typical amnestic phenotype (median age 71; median MMSE 23). Neuropsychological tests, 3D isotropic T1 MRI, [^18^F]FDG PET, and [^18^F]FBB PET (consisting of 3 early frames and 1 late frame) were performed for each subject. Structural and functional images underwent spatial registration, segmentation, and intensity normalization, using a custom Python pipeline based on FreeSurfer and ANTs tools. Basic statistics were employed to establish functional patterns and assess their statistical significance. Structural connectomes derived from probabilistic tractography on DWI images of 30 healthy subjects were employed.

**Result:**

Results revealed a significant preservation of the hippocampus in atypical AD phenotypes (p<0.05). The connectome analysis demonstrated variations in interconnections among late‐phase amyloid PET uptake regions, distinguishing between atypical and typical AD forms. Additionally, there is support for the hypothesis that early‐phase amyloid PET serves as a reliable marker for synaptic dysfunction.

**Conclusion:**

In conclusion, these preliminary findings suggest that structural connectomes in PET imaging can unveil unique neurodegenerative pathways in atypical AD patients. Moreover, the study supports the notion that early amyloid PET phases can provide insights into neurodegeneration among patients with atypical AD, akin to [^18^F]FDG PET.

